# Adrenal Androgen Predictive Effects on Clinical and Metabolic Abnormalities of Polycystic Ovary Syndrome

**DOI:** 10.1055/s-0041-1741030

**Published:** 2022-02-25

**Authors:** Sebastião Freitas de Medeiros, Bruna Barcelo Barbosa, Matheus Antônio Souto de Medeiros, Ana Karine Lin Winck Yamamoto, Márcia Marly Winck Yamamoto

**Affiliations:** 1 Department of Gynecology and Obstetrics, Medical School, Universidade Federal do Mato Grosso, Cuiabá, MT, Brazil; 2 Instituto Tropical de Medicina Reprodutiva, Cuiabá, MT, Brazil; 3 Universidade de Cuiabá, Cuiabá, MT, Brazil

**Keywords:** polycystic ovary syndrome, hyperandrogenism, obesity, hyperinsulinemia, metabolism, síndrome dos ovários policísticos, hiperandrogenismo, obesidade, hiperinsulinemia, metabolismo

## Abstract

**Objective** To examine the possible effects of adrenal prohormones in the prediction of clinical and metabolic abnormalities in women with polycystic ovary syndrome (PCOS).

**Methods** The present study enrolled 299 normal cycling non-PCOS, 156 normoandrogenemic, and 474 hyperandrogenemic women with PCOS. Baseline characteristics were compared using a chi-squared test or analysis of variance (ANOVA) as appropriate. The roles of adrenal prohormones and their ratios with total testosterone in predicting co-occurring morbidities in women PCOS were evaluated using univariate and multivariate logistic regression analyses.

**Results** Adrenal hyperandrogenism per dehydroepiandrosterone sulfate (DHEAS) levels were found in 32% of women with PCOS. In non-PCOS women, dehydroepiandrosterone (DHEA) and its sulfate had no predictive role concerning clinical, anthropometric, and metabolic parameters. In PCOS women, mainly in the hyperandrogenemic group, DHEA showed to be a significant predictor against most anthropometric-metabolic index abnormalities (odds ratio [OR] = 0.36–0.97; *p* < 0.05), and an increase in triglycerides (TG) levels (OR = 0.76; *p* = 0.006). Dehydroepiandrosterone sulfate presented a few predictive effects regarding PCOS-associated disorders. In controls, DHEAS predicted against the increase in estimated average glucose (OR= 0.38; *p* = 0.036). In the normoandrogenic group, it predicted against elevation in the waist/hip ratio (WHR) (OR= 0.59; *p* = 0.042), and in hyperandrogenemic PCOS women, it predicted against abnormality in the conicity index (CI) (OR = 0.31; *p* = 0.028).

**Conclusion** Dehydroepiandrosterone was shown to be a better predictor of abnormal anthropometric and biochemical parameters in women with PCOS than DHEAS. Thus, regarding adrenal prohormones, DHEA measurement, instead of DHEAS, should be preferred in PCOS management. The effects of androgen prohormones on the prediction of PCOS abnormalities are weak.

## Resumo

**Objetivo** Examinar os possíveis efeitos dos pró-hormônios adrenais na predição de alterações clínicas e metabólicas em mulheres com síndrome dos ovários policísticos (SOP).

**Métodos** O presente estudo envolveu 299 mulheres com ciclos menstruais regulares e 630 mulheres com SOP, sendo 156 normoandrogenêmicas e 474 hiperandrogenêmicas. As variáveis incluídas como objeto do estudo foram comparadas entre os grupos usando o teste de qui-quadrado ou análise de variância (ANOVA, na sigla em inglês). Os impactos dos pró-hormônios adrenais e suas razões com a testosterona total na predição de comorbidades em mulheres com SOP foram determinados por regressão logística univariada e multivariada.

**Resultados** Hiperandrogenismo adrenal foi encontrado em 32% das mulheres com SOP. Nos controles, a dehidroepiandrosterona e seu sulfato (DHEAS) não mostraram significância na predição das alterações clínicas, antropométricas e metabólicas. Em mulheres com SOP, principalmente no grupo de mulheres com hiperandrogenemia, a dehidroepiandrosterona (DHEA) mostrou ser um preditor significante da maioria das anormalidades nos índices antropométrico-metabólicos (odds ratio [OR] = 0,36–0,97; *p* < 0,05) e aumento nos níveis de triglicerídeos (TG) (OR = 0,76; *p* = 0,006). A DHEAS apresentou ter pouco valor na predição dos distúrbios associados à SOP; nas mulheres com androgênios elevados, restringiu-se à predição da elevação do índice de conicidade (IC) (OR = 0,31; *p* = 0,028).

**Conclusão** A DHEA mostrou ser um melhor preditor na identificação das alterações dos parâmetros antropométricos e bioquímicos em mulheres com SOP do que o seu sulfato. Assim, em relação aos pró-hormônios adrenais, a dosagem de DHEA, em vez de DHEAS, parece ser mais útil no manejo da SOP. O papel dos pró-hormônios adrenais na predição de anormalidades antropométricas e metabólicas da SOP é limitado.

## Introduction

Polycystic ovary syndrome (PCOS) is found in up to 20% of women of reproductive age. 1 Since 1990, PCOS has been defined by the presence of oligoanovulation, clinical or biochemical hyperandrogenism, and/or polycystic ovary morphology (PCOM). 2 3 4 The main characteristic of PCOS appears to be hyperandrogenism because PCOS women with high levels of androgens have increased risk for the development of central obesity, dysfunctional adipocyte, impaired fasting glucose (IFG), glucose intolerance (GI), insulin resistance (IR), dyslipidemia, metabolic syndrome (MS), low-grade chronic inflammation, nonalcoholic fatty liver disease (NAFLD), nonalcoholic steatohepatitis (NASH), and, in the end, cardiovascular disease (CVD). 5 6 7 8 9 10 All these harmful co-occurring conditions, to a certain extent, are associated with hyperandrogenemia. 11 12 13 14

Strong androgens, such as testosterone (T), direct macrophages toward adipocytes, induce adipocyte hypertrophy, promote visceral obesity, decrease adipocyte sensitivity to insulin, and decrease adipocyte glucose uptake. 15 16 17 18 19 Conversely, it appears that the adrenal prohormones dehydroepiandrosterone (DHEA) and dehydroepiandrosterone sulfate (DHEAS) have a beneficial effect by inhibiting the proliferation and differentiation of subcutaneous adipocyte and adipogenesis in omental adipocyte and enhancing adipocyte glucose uptake. 20 21 22 Furthermore, DHEA seems to exert antiglucocorticoid action on preadipocyte proliferation and differentiation, and DHEAS stimulates lipolysis. 23 24

Different effects of adrenal prohormones on the development of harmful subphenotypes in PCOS, when compared with the effects of testosterone and free testosterone index, have been reported. 25 26 The present study expands and amplifies the initial findings by examining separately women with PCOS who were classified as normoandrogenemic (NA-PCOS) and hyperandrogenemic (HA-PCOS) and comparing them with non-PCOS normal-weight controls. Therefore, the present study proposed to examine the possible beneficial effects of adrenal prohormones in preventing clinical abnormalities in women with PCOS. The secondary objective was to verify the influence of adrenal products on carbohydrate and lipid metabolism biomarkers.

## Methods

### Subjects, Design, Eligibility Criterion

The present cross-sectional study enrolled 630 women with PCOS, aged 27.6 ± 5.4 years old, and 299 normal cycling nonobese controls, aged 29.9 ± 4.8 years old ( *p* < 0.001), in whom DHEA and DHEAS were measured; all of them were attended at the outpatient clinic of the Instituto Tropical de Medicina Reprodutiva and at the Hospital Universitário Julio Muller, Cuiabá, state of Mato Grosso, Brazil, between 2003 and 2019. Women with PCOS were further divided into 156 (24.8%) NA-PCOS and 474 (75.2%) HA-PCOS. These subjects were enrolled from a previously described large sample of women with PCOS. 27 Every patient gave full-informed written consent by signing a form approved by the local Ethics in Research Committee (decision No.093/FCM/03). Late-onset adrenal enzyme deficiencies were excluded as follows: 21-hydroxylase (17-hydroxyprogesterone [17-OHP4] levels ≤ 15 nmol/L), 3β-hydroxysteroid dehydrogenase (3β-HSD) (17-hydroxypregnenolone [17-OHPE] ≤ 13.5 nmol/L), and 11-hydroxylase (compound S ≤ 23 nmol/L). Hypothyroidism was excluded by thyroid-stimulating hormone (TSH) level ≤ 4.2 µUI/mL, free thyroxin (FT4) ≤ 9.0 pmol/L, and hyperprolactinemia by prolactin (PRL) ≤ 1.1 pmol/L. 9 28 Women with PCOS who had used sex steroids, insulin-sensitizing, or dipeptidyl peptidase-4 inhibitors over the previous 6 months or those who did not fulfill the Rotterdam criteria were excluded.

### Definitions

Polycystic ovary syndrome was diagnosed using the Rotterdam criteria, after exclusion of other hyperandrogenic conditions. 28 The normal menstrual cycle was defined by a menstrual interval of between 24 and 35 days; amenorrhea was defined by the absence of a menstrual period for ≥ 90 days. 29 Frequent menses were defined as an interval < 24 days or as > 4 menstrual episodes in 90 days, and infrequent menses were defined as a menstrual cycle ≥ 35 days or as ≤ 8 menstrual periods in the previous year. 30

Because of inconsistent results due to ethnicity and inter-rater evaluation, clinical hyperandrogenism was registered as a dichotomous variable according to the complaints of the patient and the presence or lack of hirsutism in the upper lip, chin, chest, upper or lower back, upper or lower abdomen, upper arms, and thighs in the medical examination. 31 32 33 Biochemical hyperandrogenism was defined by at least 1 of the following criteria: total T ≥ 1.75 nmol/L, free testosterone (FT) ≥ 0.032 pmol/L, DHEAS ≥ 6.6 μmol/L, androstenedione (A4) ≥ 9.4 nmol/L, DHEA ≥34 nmol/L, and free androgen index (FAI) ≥ 5.2%. All these cutoff values were ≥ 90 ^th^ percentile of 425 normal cycling, normal weight, non-PCOS women. 33

Impaired fasting glucose (IFG) was defined by fasting plasma glucose concentration > 100 mg/dL (5.5 mmol/L) or < 126 mg/dL (7.0 mmol/L). Glucose intolerance (GI) was defined by a glucose concentration ≥ 7.8 nmol/L at 120 minutes after the ingestion of dextrose. Insulin resistance (IR) was defined by fasting insulin levels > 90 pmol/L and/or a HOMA-IR value ≥ 2.6. 33 Type 2 diabetes mellitus (T2DM) was defined as fasting plasma glucose ≥ 126 mg/dL (7.0 mmol/L) or glucose ≥ 200 mg/dL (11.1 mmol/L) at 120 minutes after the ingestion of dextrose. 34

### Ultrasound Evaluation

Ovarian morphology was examined by ultrasonography using a vaginal transducer with a frequency of between 5 and 8 MHz (Toshiba Xario SSA-660A, Toshiba Medical do Brazil LTDA, Taboão da Serra, SP, Brazil or Voluson E8, GE Health Care, Bedford, United Kingdom). Antral follicle count ≥ 20 follicles in at least 1 ovary and ovarian volume ≥ 10 cm3 were the reference for assuming PCOM. The ovarian volume was calculated by the ellipsoid formula: π/6 × D1 × D2 × D3, where D1, D2, and D3 were taken as the maximum diameters. 29

### Clinical and Anthropometric Parameters

All data were registered in a template used in both institutions; however, in some participants, a few observations completely at random were not recorded. Systolic blood pressure (SBP) and diastolic blood pressure (DBP) were measured with the study participants in a sitting position after at least 5 minutes of resting. Height was measured using a harpenden stadiometer (Holtain Ltd., Crymych, Dyfed, UK). Body weight was acquired using an electronic scale, and the waist circumference (WC) was measured as the narrowest measuring midway point between the lower rib margin and the iliac crest, and the hip was measured at the widest circumference at the greater trochanters. Body mass index (BMI) was calculated from the ratio between weight and height squared, despite the limitations of indirect measurement. Lean body mass (LBM) was calculated using the James 35 equation. Fat mass (FM) was calculated as body weight minus LBM. Body shape index (BSI) was calculated according to the formula: WC(cm) / [BMI ^2/3^ x height(cm) ^1/2^ ], 36 and the waist circumference triglyceride index (WTI) was calculated as the product of WC (cm) multiplied by TG (mmol/L). 37 The conicity index (CI) was calculated by the equation: WC(cm) / 10.109 × square root of BW(kg) / higher(m). 38 The VAI was calculated using the equation: WC / [36.58 + (1.89 x BMI)] x (TG / 0.81) x (1.52 / HDL-C), 39 and the lipid accumulation product (LAP) was calculated as (WC(cm)-58) x (TG9mmol)), as established for women. 40

### Biochemical Measurements

A glucose oxidase technique (Beckman Glucose Analyses, Fullerton, CA, USA) was used to measure fasting glucose. For the oral glucose tolerance test (OGTT), blood samples were collected at 0, 30, 60, 90, 120, and 180 minutes after the ingestion of 75 g of dextrose for the measurement of plasma glucose and insulin levels. 41 Glycated hemoglobin was measured using the turbidimetric assay (Wiener Laboratories, Rosario, Argentina). The estimated average glucose (eAG, mmol/L) was calculated using the equation eAG= 1.5944 multiplied by glycated hemoglobin minus 2.5944. 42 Homeostatic Model Assessment of Insulin Resistance (HOMA-IR), and basal insulin release (β-cell function, HOMA % B) were calculated using the free HOMA 2 calculator software (Diabetes Trials Unit, Oxford, UK). 43 Triglycerides (TG), high-density lipoprotein cholesterol (HDL-C), and total cholesterol (TC) were measured after a 12-hour overnight fast using an enzymatic assay (Wiener Laboratories, Rosario, Argentina). Low-density lipoprotein cholesterol (LDL-C) was calculated as TC – (HDL-C + TG/5). 44

### Hormone Measurements

Without the requirement of any resting period, blood samples were taken by cubital venipuncture between 7:30 and 10:00 am , after 10 to 12 hours of fasting, between the 3 ^rd^ and 5 ^th^ days of spontaneous menstruation or, in the case of infrequent menses or amenorrhea, on any day regardless of the time elapsed since the last menstrual period; in this case, the progesterone (P4) level was used to certify that samples were collected in the follicular phase and the results were validated whenever the P4 level was < 6.3 nmol/L. 45 46 Thyroid-stimulating hormone, estradiol (E2), PRL, SHBG, total T, DHEA, and FT4 were measured with an electrochemiluminescence assay (Elecsys 1010, Roche Diagnostics GmbH, Mannheim, Germany). Serum P4, A4, DHEAS, cortisol (F), and insulin were measured using a chemiluminescence assay (Advia Centaur, Siemens Healthcare Diagnostics, UK or Siemens Medical Solution Diagnostics, Los Angeles, CA, USA). Free testosterone concentrations were measured using an ELISA kit (GenWay Biotech Inc., San Diego, CA, USA). 17-hydroxyprogesterone levels were verified using a coat-a-count radioimmunoassay (Siemens Health Care Diagnostics Inc., Los Angeles CA, USA). The precision of these hormone measurements was verified in a recent publication. 33 The FAI was calculated as T (nmol/L divided by SHBG (nmol/L) × 100, and index of hyperandrogenism (IHA) was estimated as fifth root FAI x A4 X DHEA x DHEAS. 46

### Statistical Analysis

The original data of each variable were initially submitted to the Grubs test to avoid interference of outliers. Distributions of all variables were examined using the Shapiro–Wilk test. Data with asymmetric distribution are presented as numbers and percentages (n%), and data with symmetric distribution are shown as mean (x̅) and standard deviation (SD). Proportions were compared using the chi-squared test or the Fisher exact test when appropriate. Comparisons of Gaussian variables were performed using one-way analysis of variance (ANOVA). Univariate logistic regression was applied to examine the relationship between anthropometric and metabolic parameters as dependent variables and DHEA, DHEAS, DHEA/T ratio, and DHEAS/T ratios as independent variables. The strength of independent variables in predicting anthropometric and metabolic abnormalities were controlled by age and BMI and given as odds ratio (OR) and 95% confidence interval (CI). The Kruskal-Wallis test, followed by the Bonferroni post-hoc test, was used in univariate logistic regression. For all univariate logistic regression analyses, the 90 ^th^ percentile of dependent variables, taken from 425 normal-weight non-PCOS controls, was used as a cutoff. 9 The explained variation in the criterion variable was given by Cox and Snell *R ^2^* and Nagelkerke *R ^2^* values. Multivariate forward stepwise logistic regression was done including DHEA, DHEAS, DHEA/T, and DHEAS/T as significant independent variables in the models. Age and BMI were also included to control for confounders. The fit of the logistic regression models was evaluated using the Hosmer–Lemeshow goodness-of-fit test. Receiver operating characteristic (ROC) analysis curves were calculated using DHEA, DHEAS, DHEA/T, and DHEAS/T as independent variables, and significant anthropometric and metabolic variables were used as dependent variables. All tests were two-sided, and p-values < 0.05 were considered statistically significant. All statistical procedures were performed using SPSS Statistics for Windows, version 17 (SPSS Inc, Chicago, IL, USA).

## Results

Comparisons of age, ethnicity, clinical, and hormone parameters among groups are shown in Table 1 . Most PCOS patients were Caucasians, 72.4% NA-PCOS and 70.6% HA-PCOS ( *p* = 0.603). African descendants were 9.6 and 12.7% NA-PCOS and HA-PCOS, respectively ( *p* = 0.307). Women of other ethnicities were also similar in NA-PCOS and HA-PCOS (18.0 versus 16.0%, respectively, *p* = 0.711). Among controls, the distribution of these ethnicities was different from that of the PCOS groups: 89.3% were Caucasians, 60.0% were African descendants, and 4.7% were of other races ( *p* < 0.05 for all comparisons). Comparisons of anthropometric, anthropometric-metabolic indexes, and metabolic characteristics of normal cycling controls, NA-PCOS, and HA-PCOS women are shown in Supplementary Table S1 (available online only).

**Table 1:** Comparisons of baseline clinical characteristics of normoandrogenemic, hyperandrogenemic, women with polycystic ovary syndrome, and normal cycling controls

Variables	Non-PCOS	NA-PCOS	HA-PCOS
Age (years old)	** 29.9 ± 4.8 ^a^**	** 29.0 ± 5.2 ^a^**	** 27.0 ± 5.5 ^b^**
Ethnicity (n,%) *			
Caucasian	267 (89.3) ^a^	113 (72.4) ^b^	335 (70.6) ^b^ , **
African descendent	18 (6.0) ^a^	15 (9.6) ^a^	60 (12.7) ^b^
Other	14 (4.7) ^a^	28 (18.0) ^b^	79 (16.7) ^b^
Blood pressure ( *x̅* ± SD) ***			
SBP (mmHg)	113.1 ± 9.5 ^a^	116.6 ± 72.1 ^b^	120.0 ± 12.4 ^b^
DBP (mmHg)	71.4 ± 9.0 ^a^	74.6 ± 9.3 ^b^	76.2 ± 9.3 ^b^
Clinical signs (n,%) *			
Acne	12 (4.0) ^a^	70 (44.9) ^b^	217 (45.7) ^b^
Hirsutism	7 (2.3) ^a^	72 (46.2) ^b^	224 (47.3) ^b^
Acanthosis	2 (0.7) ^a^	27 (17.3) ^b^	95 (20.0) ^b^
Striae	0 (0.0) ^a^	19 (12.2) ^b^	70 (14.7) ^b^
Galactorrhea	0 (0.0) ^a^	2 (1.3) ^b^	6 (1.3) ^b^
Metabolic disorders *			
IFG	8 (2.6) ^a^	8 (5.1) ^b^	62 (13.1) ^c^
GI	11 (3.7) ^a^	16 (6.9) ^b^	78 (26.3) ^c^
T2DM	10 (3.3) ^NS^	5 (5.0) ^NS^	18 (6.0) ^NS^
Hormones *			
DHEAS > 34nmol/l	32 (10.7) ^a^	8 (5.1) ^a^	32 (6.8) ^a^
DHEAS > 6.6µl/l	29 (9.7) ^a^	1 (0.6) ^a^	144 (31.8) ^b^
T > 1.75 mmol/l	30 (10.0) ^a^	11 (7.1) ^a^	328 (70.4) ^b^
FAI > 5.2	12 (4.0) ^a^	19 (12.2) ^b^	260 (61.3) ^c^

Abbreviations: DBP, diastolic blood pressure; DHEAS, dehydroepiandrosterone sulfate; DM, diabetes mellitus; FAI, free androgen index; GI, glucose intolerance; HA-PCOS, hyperandrogenemic polycystic ovary syndrome; IFG, impaired fasting glucose; NA-PCOS, normoandrogenemic polycystic ovary syndrome; SBP, systolic blood pressure; T, testosterone.

* Comparisons using chi-squared or Fisher tests as appropriate.

** Equal subscript letter denotes proportions that do not differ significantly from each other at the level of 0.05; otherwise, different letters indicate *p* < 0.05 between proportions or means ± standard deviation. NS= not significant.

*** Comparisons using one-way analysis of variance.

### The Predictive Role of DHEA in Clinical, Anthropometric, and Metabolic Parameters

In non-PCOS controls, DHEA had no significant predictive role but tended to predict an increase in SBP (OR = 2.47; *p* = 0.054; area under the curve [AUC] = 0.705; *p* < 0.001), and a decrease in eAG (OR = 0.69; *p* = 0.051; AUC = 0.700; *p* = 0.006) without reaching statistical significance. In NA-PCOS ( Table 2 ), DHEA levels predicted elevation in body weight (OR = 2.58; *p* = 0.039), fasting glucose (OR = 4.36; *p* = 0.021), and against increase in HOMA %B (OR = 0.58; 0.029). In HA-PCOS, Table 3 , DHEA significantly predicted against the increase in most anthropometric-metabolic indexes (OR = 0.36–0.97). Regarding biochemical markers, DHEA levels predicted against elevation of TG concentrations (OR = 0.76; *p* = 0.006).

**Table 2:** Age and body mass index controlled univariate logistic regression between clinical, anthropometric, anthropometric-metabolic indexes, and metabolic biomarkers as dependent variables, and dehydroepiandrosterone as the independent variable in normoandrogenemic women with polycystic ovary syndrome

Variable	B	Wald	Sig	Exp B (Lower-Upper)	AUC	*p-value*
SBP (mmHg)	−0.424	1.761	0.184	0.655 (0.350–1.224)	0.842	< 0.001
DBP (mmHg)	−0.222	0.286	0.593	0.801 (0.355–1.808)	0.757	0.006
BW (kg)	0.950	4.247	0.039	2.584 (1.048–6.372)	0.976	< 0.001
WC (cm)	−0.206	0.385	0.535	0.814 (0.424–1.560)	0.957	< 0.001
WHR (ratio)	−0.295	0.627	0.429	0.745 (0.359–1.545)	0.697	0.019
FM (kg)	0.592	1.033	0.309	1.808 (0.577–5.667)	0.991	< 0.001
FM (%)	0.640	1.286	0.257	1.826 (0.628–5.726)	0.989	< 0.001
FM/LBM (ratio)	0.172	0.163	0.686	1.194 (0.505–2.824)	0.980	< 0.001
WTI	−0.107	0.169	0.681	0.898 (0.835–1.499)	0.789	< 0.001
CI (%,pg/ml, nmol/L)	−0.262	1.089	0.297	0.770 (0.471–1.258)	0.748	< 0.001
BSI	−0204	1.043	0.307	0.816 (0.552–1.206)	0.600	0.041
VAI	0.242	0.689	0.407	1.274(0.719–2.257)	0.734	< 0.001
LAP (cm, mmol/L)	−0.424	2.008	0.157	0.654 (0.364–1.177)	0.885	< 0.001
Go (mmol/L)	1.473	5.321	0.021	4.363 (1.248–15.257)	0.924	< 0.001
Io (pmol/L)	−0.069	3.742	0.053	0.9336 (0.820–1.001)	0.838	< 0.001
C-pep (pmol/L)	−0.455	3.182	0.074	0.635 (0.385–1.046)	0.780	< 0.001
HOMA-IR	0.095	0.089	0.766	1.100 (0.582–1.063)	0.784	< 0.001
HOMA %B	−0.544	4.740	0.029	0.581 (0.356–0.947)	0.739	< 0.001
eAG (mmol/L)	−0.075	0.089	0.765	0.928 (0.567–1.518)	0.638	0.027
TC (mmol/L)	0.010	0.001	0.971	1.010 (0.576–1.771)	0.554	0.293
HDL-C (mmol/L)	−0.025	0.793	0.373	0.975 (0.922–1.031)	0.700	< 0.001
LDL-C (mmol/L)	−0.135	0.255	0.613	0.874 (0.519–1.473)	0.552	0.431
VLDL-C (mmol/L)	0.009	0.054	0.816	1.009 (0.937–1.086)	0.732	< 0.001
TG (mmol/L)	0.025	0.567	0.452	1.026 (0.960–1.096)	0.722	< 0.001
DM	−0.072	0.517	0.472	0.931 (0.765–1.132)	0.868	0.006
IG	0.019	0.134	0.714	1.019 (0.920–1.129)	0.820	< 0.001

Abbreviations: SBP, systolic blood pressure; DBP, diastolic blood pressure; BW, body weight; WC, waist circumference; WHR, waist-hip ratio; FM, fat mass; LBM, lean body mass; WTI, waist circumference-triglyceride index; CI, conicity index; BSI, body shape index; VAI; visceral adiposity index; LAP, lipid accumulation product; Go, fasting glucose; Io, fasting insulin; C-pep, C-peptide; HOMA-IR,homeostatic assessment model of insulin resistance; HOMA%B, homeostatic model assessment of β-cell function; eAG, average glucose; TC, total cholesterol; HDL-C, high-density lipoprotein cholesterol; LDL-C, low-density lipoprotein cholesterol; VLDL-C, very-low-density lipoprotein cholesterol; TG, triglyceride; DM, diabete mellitus; IG, intolerance glucose.

**Table 3:** Age and body mass index controlled univariate logistic regression between clinical, anthropometric, anthropometric-metabolic indexes, and metabolic biomarkers as dependent variables, and dehydroepiandrosterone as the independent variable in hyperandrogenemic women with polycystic ovary syndrome

Variable	B	Wald	Sig	Exp B (Lower-Upper)	AUC	*p-value*
SBP (mmHg)	0.059	0.327	0.567	1.061 (0.866–1.300)	0.773	< 0.001
DBP (mmHg)	−0.098	0.559	0.455	0.907 (0.702–1.171)	0.751	< 0.001
BW (kg)	0.003	0.001	0.978	1.003 (0.794–1.268)	0.947	< 0.001
WC (cm)	−1.072	2.968	0.085	0.342 (1.101–1.159)	0.957	< 0.001
WHR	−0.189	2.429	0.119	0.828 (0.652–1.050)	0.730	< 0.001
FM (kg)	0.103	0.461	0.497	1.108 (0.824–1.491)	0.987	< 0.001
FM (%)	0.013	0.008	0.929	1.013 (0.767–1.336)	0.977	< 0.001
FM/LBM (ratio)	−0.005	0.001	0.976	0.995 (0.711–1.392)	0.986	< 0.001
WTI (WC/TG)	−0.227	5.256	0.022	0.797 (0.656–0.968)	0.803	< 0.001
CI (%,pg/ml, nmol/L)	−0.841	0.3178	0.049	0.451 (0.187–0.996)	0.742	< 0.001
BSI	−0.274	11.388	0.001	0.761 (0.649–0.892)	0.601	< 0.001
VAI	−0.999	4.204	0.040	0.369 (0.142–0.957)	0.726	< 0.001
LAP (cm, mmol/L)	−0.024	3.971	0.046	0.976 (0.954–1.000)	0.886	< 0.001
Go (mmol/L)	0.676	1.302	0.254	1.966 (0.616–6.282)	0.708	< 0.001
Io (pmol/L)	0.175	0.161	0.688	1.192 (0.506–2.084)	0.785	< 0.001
C-pep (nmol/L)	−0.138	1.815	0.178	0.871 (0.713–1.065)	0.832	< 0.001
HOMA-IR	0.065	0.017	0.897	1.067 (0.397–2.866)	0.774	< 0.001
HOMA %B	0.133	0.098	0.755	1.143 (0.495–2.638)	0.701	< 0.001
eAG (mmol/L)	−0.022	0.055	0.814	0.978 (0.812–1.178)	0.699	< 0.001
TC (mmol/L)	0.145	0.096	0.752	1.156 (0.461–2.898)	0.630	< 0.001
HDL-C (mmol/L)	0.442	1.094	0.296	1.556 (0.679–3.565)	0.688	< 0.001
LDL-C (mmol/L)	0.084	0.035	0.852	0.919 (0.378–2.233)	0.602	0.002
VLDL-C (mmol/L)	−0.074	0.492	0.483	0.928 (0.755–1.142)	0.698	< 0.001
TG (mmol/L)	−0.270	0.748	0.006	0.763 (0.629–0.926)	0.740	< 0.001
DM	−0.212	0.836	0.361	0.809 (0.513–1.278)	0.812	< 0.001
IG	0.175	2.265	0.132	1.192 (0.948–1498)	0.732	< 0.001

Abbreviations: SBP, systolic blood pressure; DBP, diastolic blood pressure; BW, body weight; WC, waist circumference; WHR, waist-hip ratio; FM, fat mass; LBM, lean body mass; WTI, waist circumference-triglyceride index; CI, conicity index; BSI, body shape index; VAI; visceral adiposity index; LAP, lipid accumulation product; Go, fasting glucose; Io, fasting insulin; C-pep, C-peptide; HOMA-IR,homeostatic assessment model of insulin resistance; HOMA%B, homeostatic model assessment of β-cell function; eAG, average glucose; TC, total cholesterol; HDL-C, high-density lipoprotein cholesterol; LDL-C, low-density lipoprotein cholesterol; VLDL-C, very-low-density lipoprotein cholesterol; TG, triglyceride; DM, diabete mellitus; IG, intolerance glucose.

### The Predictive Role of DHEAS in Clinical, Anthropometric, and Metabolic Parameters

In non-PCOS controls, DHEAS was not associated with any abnormality in anthropometric or anthropometric-metabolic index. Regarding metabolic parameters, DHEAS predicted against increase in eAG (OR = 0.38; *p* = 0.036; AUC = 0.706; *p* = 0.001). In NA-PCOS women, DHEAS only predicted against increase in WHR (OR = 0.59; *p* = 0.042; AUC = 0.757; *p* = 0.002). In HA-PCOS, DHEAS concentrations predicted against elevation in the CI (OR = 0.31; *p* = 0.028; AUC = 0.740; *p* < 0.001) ( Table 3 ).

### The Predictive Role of DHEA/total Testosterone Ratio in Clinical, Anthropometric, and Metabolic Parameters

The DHEA/total testosterone ratio (DHEA/T) was a good predictor against increase in WHR (OR = 0.44; *p* = 0.015; AUC = 0.662; *p* < 0.001) in the control group. In NA-PCOS women, the DHEA/T ratio tended to predict against increase in BW (OR = 0.95; *p* = 0.071; AUC = 0.974; *p* < 0.001), and decrease in C-pep levels (OR = 0.68; *p* = 0.070; AUC = 0.805; *p* < 0.001). This ratio predicted increase in fasting glucose (OR = 2.65; *p* = 0.018; AUC = 0.915; *p* < 0.001). In HA-PCOS women ( Table 4 ), the DHEA/T ratio predicted against increase in WTI (OR = 0.70; *p* = 0.003), CI (OR = 0.79; *p* = 0.036), BSI (OR = 0.77; *p* = 0.046), VAI (OR = 0.76; *p* = 0.039), and LAP (OR = 0.76; *p* = 0.032). However, with statistical significance, the DHEA/T ratio predicted against increase in TC (OR = 0.76; *p* = 0.033), in VLDL-C (OR = 0.90; *p* = 0.049), and in TG (OR = 0.72; *p* = 0.006).

**Table 4:** Age and body mass controlled univariate logistic regression between clinical, anthropometric, and anthropometric-metabolic indexes as dependent variables, and dehydrotestosterone/testosterone ratio as the independent variable in hyperandrogenic women with polycystic ovary syndrome

Variable	B	Wald	Sig	Exp B (Lower-Upper)	AUC	*p-value*
SBP (mmHg)	0.279	0.387	0.568	1.323 (0.508–3.441)	0.773	< 0.001
DBP (mmHg)	0.130	0.048	0.826	1.138 (0.357–3.627)	0.742	< 0.001
BW (kg)	0.058	0.009	0.924	1.060 (0.320–3.500)	0.947	< 0.001
WC (cm)	−0.146	0.954	0.329	0.864 (0.645–1.158)	0.957	< 0.001
WHR (ratio)	−0.195	1.779	0.182	0.823 (0.618–1.096)	0.722	< 0.001
FM (kg)	0.013	0.005	0.944	1.013 (0.709–1.447)	0.987	< 0.001
FM (%)	0.342	0.225	0.636	1.408 (0.342–5.792)	0.978	< 0.001
WTI (ratio)	−0.355	8.615	0.003	0.701 (0.553–0.889)	0.809	< 0.001
CI (%, pg/ml, nmol/L)	−0.227	4.406	0.036	0.797 (0.645–0.985)	0.739	< 0.001
BSI (ratio)	−0.252	3.988	0.046	0.777 (0.607–0.995)	0.888	< 0.001
VAI	−0.267	4.276	0.039	0.766 (0.594–0.986)	0.723	< 0.001
LAP (cm, mmol/L)	−0.275	4.624	0.032	0.760 (0.591–0.976)	0.888	< 0.001
Go (mmol/L)	−0.032	1.773	0.183	0.968 (0.923–1.015)	0.703	< 0.001
Io (pmol/L)	−0.014	0.016	0.898	0.987 (0.803–1.273)	0.783	< 0.001
HOMA-IR	−0.492	0.946	0.331	0.611 (0.227–1.648)	0.775	< 0.001
HOMA %B	0.387	0.800	0.371	1.472 (0.632–3.437)	0.694	< 0.001
C-pep (nmol/L)	−0.028	2.464	0.116	0.972 (0.938–1.007)	0.838	< 0.001
eAG (mmol/L)	−0.561	1.544	0.214	0.571 (0.235–1.303)	0.719	< 0.001
TC (mmol/L)	−0.216	4.168	0.033	0.766 (0.600–0.962)	0.652	< 0.001
HDL-C (mmol/L)	−0.046	0.204	0.652	0.955 (0.783–1.166)	0.689	< 0.001
VLDL-C (mmol/L)	−0.040	3.888	0.049	0.961 (0.923–1.000)	0.705	< 0.001
TG (mmol/L)	−0.738	7.629	0.006	0.720 (0.920–0.909)	0.740	< 0.001
LDL-C (mmol/L)	−0.115	1.007	0.316	0.891 (0.711–1.116)	0.611	< 0.001
DM	−0.091	2.495	0.114	0.913 (0.815–1.022)	0.849	< 0.001
IG	−0.059	0.010	0.918	0.943 (0.306–2.909)	0.735	< 0.001

Abbreviations: SBP, systolic blood pressure; DBP, diastolic blood pressure; BW, body weight; WC, waist circumference; WHR, waist-hip ratio; FM, fat mass; WTI, waist circumference-triglyceride index; CI, conicity index; BSI, body shape index; VAI; visceral adiposity index; LAP, lipid accumulation product; Go, fasting glucose; Io, fasting insulin; HOMA-IR, homeostatic assessment model of insulin resistance; HOMA%B, homeostatic model assessment of β-cell function; C-pep, C-peptide; eAG, average glucose; TC, total cholesterol; HDL-C, high-density lipoprotein cholesterol; VLDL-C, very-low-density lipoprotein cholesterol; TG, triglyceride; LDL-C, low-density lipoprotein cholesterol; DM, diabete mellitus; IG, intolerance glucose.

### The Predictive Role of DHEAS/Total Testosterone Ratio in Clinical, Anthropometric, and Metabolic Parameters

In non-PCOS controls ( Table 5 ), the DHEAS/T ratio predicted only against the increase in WHR (OR = 0.44; *p* = 0.015; AUC = 0.663; *p* < 0.001). In NA-PCOS, the DHEAS/ T ratio did not predict any biomarker of anthropometric or metabolic abnormalities. In the HA-PCOS group, the DHEAS/T ratio predicted against increase in CI (OR = 0.29; *p* = 0.007; AUC = 0.738; *p* < 0.001), and in TC (OR = 0.37; *p* = 0.041; AUC = 0.655; *p* = 0.001). Without reaching statistical significance, this ratio also tended to predict against increase in VLDL-C (OR = 0.87; *p* = 0.084; AUC = 0.570; *p* < 0.001), and in WTI (OR = 0.64; *p* = 0.067; AUC = 0.719; *p* < 0.001).

**Table 5:** Final models of multivariate logistic regression analysis between anthropometric, anthropometric-metabolic, and metabolic indexes it is dependent variables and androgens as independent variables in non-polycystic ovary syndrome controls and women with polycystic ovary syndrome

Dependent variables	Retained independent variables	B	Wald	P	Exp B (Lower-Upper)	AUC	*p-value*
Non-PCOS							
WHR (ratio)	DHEAS/T	−0.8113	5.857	0.016	0.444(0.230–0.857)	0.663	< 0.001
eAG (mmol/L)	DHEAS	−1.881	4.848	0.028	0.152(0.145–0.903)	0.704	0.001
NA-PCOS							
BW (kg)	DHEA	0.979	4.360	0.037	2.662(1.062–6.672)	0.977	< 0.001
Go (mmol/L)	DHEA/T	0.957	5.498	0.019	2.605(1.170–5.800)	0.906	< 0.001
HOMA %B	DHEA	−0.543	4.277	0.039	0.581(0.348–0.972)	0.739	< 0.001
HA-PCOS							
WTI (ratio)	DHEA/T	−0.350	8.263	0.004	0.705(0.555–0.895)	0.809	< 0.001
CI (%, pg/ml, nmol/L)	DHEAS/T	0.434	8.405	0.004	1.544(1.151–2.071)	0.759	< 0.001
VAI	DHEA	−0.299	7.511	0.006	0.741(0.599–1.136)	0.719	< 0.001
LAP (cm, mmol/L)	DHEA/T	−0.265	4.291	0.038	0.767(0.597–0.986)	0.888	< 0.001
TC (mmol/L)	DHEAS/T	−1.189	8.037	0.005	0.305(0.134–0.693)	0.666	< 0.001
TG (mmol/L)	DHEA	−0.300	9.380	0.002	0.741(0.611–0.897)	0.739	< 0.001

Abbreviations: Non-PCOS, not polycystic ovary syndrome; WHR, waist-hip ratio; eAG, average glucose; NA-PCOS, normoandrogenemic polycystic ovary syndrome; BW, body weight; Go, fasting glucose; HOMA%B, homeostatic model assessment of β-cell function; HA-PCOSβ hyperandrogenemic polycystic ovary syndrome; WTI, waist circumference-triglyceride index; CI, conicity index; VAI; visceral adiposity index; LAP, lipid accumulation product; TC, total cholesterol; TG, triglyceride.

### Multivariate Logistic Regression Analysis

Multivariate logistic regression models, consistent with univariate regression, retained the predictive role of the DHEAS/T ratio against increase in WHR (OR = 0.44; *p* = 0.016) and of DHEAS against increase in eAG (OR = 0.36; *p* = 0.029) in the non-PCOS control group. In NA-PCOS women, the results of the univariate regression were maintained after multiple regression. Thus, DHEA maintained the predictive role favoring increase in BW (OR = 2.60; *p* = 0.019). In these women, DHEA also predicted against the increase in HOMA %B (OR = 0.34; *p* = 0.039). The DHEA/T ratio favors an increase in fasting glucose (OR = 2.60; *p* = 0.019). In HA-PCOS, DHEA maintained prediction against the increase in VAI (OR = 0.74; *p* = 0.006) and in TG (OR = 0.74; *p* = 0.002). The DHEAS/T ratio predicted against the increase in TC (OR = 0.30; *p* = 0.005); nevertheless, the DHEAS/T ratio favors an increase in CI (OR = 1.54; *p* = 0.004). The DHEA/T ratio predicted against an increase in WTI (OR = 0.70; *p* = 0.004) and in LAP (OR = 0.76; *p* = 0.038).

## Discussion

Many studies have investigated the role of adrenal androgens in PCOS. There have been attempts to translate basic knowledge into the clinical practice. The present study evaluated the strength of adrenal androgens in predicting blood pressure, anthropometric, and metabolic abnormalities in women with PCOS, after separating PCOS women with normal from high androgens in the blood. The impact of DHEA, DHEAS, and the ratios of these prohormones and total testosterone were considered because between ∼ 70 and 95% of DHEA and DHEAS molecules have the adrenal gland as a primary source. 26 Either in a combination of various androgens or in the elevation of at least one androgen, biochemical hyperandrogenism is found in > 70% of women with PCOS. 46 47 Higher androgens of the adrenal source are reported in between 20 and 30% of PCOS women. 48 49 Insulin resistance is a common comorbidity of hyperandrogenemic PCOS through decreased insulin-like growth factor 1 (IGF-BP) leading to increased free insulin-like growth factor 1 (IGF-1), which stimulates ovarian androgen production. 50 The role of insulin resistance in the adrenal function of PCOS women is unclear. 51 Conversely, the cross-talk between adrenal androgens and insulin acting in women with PCOS is of clinical relevance. Furthermore, while insulin is higher in obesity, cortisol (F), DHEA, and DHEAS appear to be lower. 52

The incidence of 75% of biochemical hyperandrogenism in women with PCOS in the present study is in agreement with other studies. 53 54 55 As shown in Table 1 , the slightly higher SBP and DBP levels in PCOS than in controls, observed mainly in the hyperandrogenemic group, found in the present study, also endorse previous findings. 56 57 58 In the same way, a higher prevalence of glucose intolerance in women with PCOS has been extensively found. 7 13 45 59 60 The finding that, in NA-PCOS, DHEA was negatively correlated with body weight, fasting glucose, and β-cell activity, indicates a protective role of DHEA against adiposity and insulin resistance. In previous studies, DHEA has been shown to inhibit the proliferation and differentiation of adipocytes in the subcutaneous adipose tissue compartment (SAT) and to increase adipocyte glucose uptake in this location. 21 61 Additionally, DHEA appears to inhibit adipogenesis in omental adipocytes through an increase in resistin production. 20 62 Dehydroepiandrosterone also increases insulin signaling to its secretion and protects against omental adipogenesis. 63 Furthermore, DHEA suppresses the activity and expression of glucose-6-phosphate and of phosphoenol carboxykinase, decreasing gluconeogenesis and increasing glucose uptake in the adipocyte and hepatocyte. 64 Clinical biomarkers of central adiposity were not correlated with DHEA concentrations in the present study in normoandrogenemic women with PCOS.

On the other hand, in HA-PCOS women, DHEA was negatively correlated with various anthropometric-metabolic indexes and TG abnormalities. Thus, the present study supports the knowledge of the beneficial role of DHEA levels in women with PCOS, at least in those with high androgen levels. 18 65 Both in vitro and in vivo studies have shown a protective role of DHEA in the cardiovascular system. 66 Furthermore, lower levels of DHEA are associated with increased body fat accumulation. 67 In contrast, high levels of DHEA are associated with lower BMI, 68 lower body fat accumulation, and lower risk of T2DM. 69 70 An antiatherogenic effect of DHEA through inhibition of fibroblast growth, improvement of lipid profile, and decrease of platelet aggregation has also been shown. 71 It is worthy of note that lower levels of DHEA are found in hyperinsulinemic status due to its diminished synthesis or increased metabolic clearance. Additionally, high levels of glucose and insulin might impair DHEA synthesis in the adrenal gland. 72

In PCOS, as a whole group, high levels of DHEAS have been found in between 18 and 72%. 25 59 73 74 The high levels of DHEAS have also been associated with a favorable metabolic and cardiovascular profile. 74 75 76 77 78 DHEAS opposes T action concerning the risk of obesity, and IR. 25 High levels of DHEAS are associated with lower BMI 68 when compound with controls and its levels decrease after weight loss after bariatric surgery. 79 Dehydroepiandrosterone sulfate levels have been negatively correlated with WC, WHR, LDL-C, and TG concentrations, adjusted for the confounding effects of age and BMI. 74 78 Furthermore, DHEAS levels were negatively correlated with carotid intima-media thickness 75 and improved endothelial function. 80 The predictive value of DHEAS against abnormalities in WHR and in the CI of women with PCOS seen in the present study supports its beneficial effect on predicting anthropometric and metabolic derangements. 25 74 81 82

The DHEA/T ratio was reported to be strongly associated with insulin sensitivity. 63 However, there is an inconsistent correlation between the DHEAS/T ratio, insulin levels, and insulin receptor binding. 83 In the clinical practice, this ratio has been used for the prediction and early diagnosis of metabolic syndrome and it appears to antagonize the effect of T in women with PCOS, which is associated with a favorable metabolic profile. 25 84 Furthermore, this ratio has been negatively correlated with lower BMI, lower WC, lower WHR, lower TG, lower LDL-C, and higher HDL-C, insulin levels, and HOMA-IR. 76 77 78 It also appears that the DHEA/T ratio improves lipid metabolism. 84 85 86 87 88 The present study supports the knowledge that the DHEA/T ratio predicts various abnormalities in anthropometric-metabolic indexes in PCOS women with biochemical hyperandrogenism and against the increase in fasting glucose despite normal androgens in the blood. Nevertheless, the predictive effect of DHEAS/T against anthropometric and metabolic abnormalities in PCOS is limited.

A few limitations must be considered in the analysis of the present study. The cross-sectional design does not allow to determine causal effects; instead, it provides associations or predictions. The assays used may have some imprecision, but they have presented a good correlation with the gold-standard high-performance liquid chromatography-tandem mass spectrometry assays. 89 90 The sample size, a clear definition for normoandrogenemic and hyperandrogenemic groups of women with PCOS, and their analysis in separate are the principal strengths of the present study.

## Conclusion

Dehydroepiandrosterone has been demonstrated to be a better predictor of abnormal anthropometric and biochemical parameters in women with PCOS than DHEAS, particularly in hyperandrogenemic women. The DHEA/T ratio has also shown increased prediction to predict against increase in anthropometric and metabolic parameters in PCOS. Dehydroepiandrosterone measurement seems to be preferred in PCOS management. In general, in the clinical practice, it must be highlighted that the predictive and protective effects of both adrenal hormones DHEA and DHEAS are mild or weak.
