# Supplementary material for: Adrenal Androgen Predictive Effects on Clinical and Metabolic Abnormalities of Polycystic Ovary Syndrome
Source: Rev Bras Ginecol Obstet. 2022 Feb 25;44(2):142–53. doi: 10.1055/s-0041-1741030 (PMC9948267; doi:10.1055/s-0041-1741030)
Supplement: Supplementary file 1 — Supplementary Material [file 10-1055-s-0041-1741030-s210028.pdf]

Supplementary Table S1 Comparisons of baseline anthropometric and biochemical characteristics of normoandrogenemic and hyperandrogenemic polycystic ovary syndrome and normal cycling controls

Variables*	Non-PCOS ($\bar{x} \pm$ SD)	NA-PCOS ($\bar{x} \pm$ SD)	HA-PCOS ($\bar{x} \pm$ SD)
Anthropometric			
BW (kg)	61.4 ± 8.8	70.8 ± 16.7	73.4 ± 17.0[a,b]
BMI (kg/m^2)	23.4 ± 3.0	27.5 ± 1.2	28.6 ± 6.6[a,b]
DBP (mmHg)	116.9 ± 9.5	116.9 ± 12.4	118.7 ± 12.1[a,b]
SBP (mmHg)	71.9 ± 9.0	74.6 ± 6.0	76.1 ± 9.3[a,b]
WC (cm)	72.4 ± 7.1	82.4 ± 1.6	85.1 ± 1.1[a,b,c]
WHR (ratio)	0.74 ± 0.05	0.78 ± 0.07	0.81 ± 0.08[a,b,d]
LBM (kg)	43.2 ± 4.4	46.3 ± 5.9	46.5 ± 6.6[a,b]
LBM (%)	70.8 ± 5.9	65.0 ± 7.6	63.5 ± 8.4[a,b]
FM (kg)	18.3 ± 5.9	25.5 ± 1.1	27.4 ± 1.3[a,b]
FM (%)	29.9 ± 5.8	34.9 ± 7.6	35.8 ± 8.4[a,b]
FM/LBM (ratio)	0.42 ± 0.1	0.56 ± 0.2	0.60 ± 0.2[a,b]
Anthropometric metabolic indexes			
WTI (WC/TG)	60.6 ± 4.4	83.1 ± 8.7	115.3 ± 13.9[a,b,d]
CI (%,pg/ml,nmol/L)	1.07 ± 0.06	1.12 ± 0.01	1.16 ± 0.01[a,b,e]
BSI (10^{-}m$^{11/6}$kg$-^{2.3}$)	0.19 ± 0.01	0.21 ± 0.02	0.22 ± 0.03
VAI (cm, kg,/m, mmol)	1.08 ± 0.08	1.71 ± 0.02	1.96 ± 0.02[a,b]
LAP (cm, mmol/L)	11.8 ± 2.1	21.8 ± 2.5	29.5 ± 2.7[a,b,f]
Metabolic parameters			
Go (mmol/L)	4.7 ± 0.4	4.8 ± 0.4	5.0 ± 0.6[b,f]
G120 (mmol/L)	5.2 ± 1.1	6.5 ± 1.9	6.6 ± 2.2[b,g]
Io (pmol/L)	43.7 ± 3.2	76.7 ± 6.7	81.2 ± 9.3[a,b,d]
Go/Io (ratio)	0.11 ± 0.01	0.07 ± 0.01	0.07 ± 0.04[a,b]
HOMA-IR	0.87 ± 0.01	1.41 ± 0.11	1.70 ± 0.16[a,b,d]
HOMA%B	95.3 ± 3.3	125.8 ± 1.4	128.8 ± 6.3[a,b]
C-pep (nmol/L)	0.49 ± 0.02	0.74 ± 0.40	0.83 ± 0.41[a,b,h]
eAG (mmol/L)	5.3 ± 0.6	5.8 ± 1.4	6.0 ± 1.3[a,b]
TC (mmol/L)	4.3 ± 0.8	4.6 ± 0.7	4.6 ± 0.9[b,i]
HDL-C (mmol/L)	1.4 ± 1.2	1.2 ± 0.3	1.2 ± 0.3[a,b]
LDL-C (mmol/L)	2.5 ± 0.7	2.7 ± 0.8	2.8 ± 0.7[b,j]
VLDL-C (mmol/L)	0.44 ± 0.03	0.58 ± 0.37	0.65 ± 0.46[b,k]
TG (mmol/L)	0.85 ± 0.05	1.20 ± 0.67	1.40 ± 0.89[b,l,m]
Hormones			
T (nmol/L)	1.0 ± 0.2	1.0 ± 0.3	2.0 ± 0.1[a,b]
FT pmol/L)(	0.011 ± 0.001	0.013 ± 0.001	0.042 ± 0.001[a,b,n]
DHEA (nmol/L)	14.2 ± 1.9	13.6 ± 0.9	16.4 ± 1.6[e,i,n]
DHEAS (μmol/l)	3.8 ± 0.2	3.5 ± 0.2	5.3 ± 0.3[b,n]
A4 (nmol/L)	4.9 ± 0.4	5.1 ± 0.5	10. ± 0.6[b,n]
SHBG (nmol/L)	60.4 ± 3.2	49.7 ± 3.3	33.1 ± 3.2[a,b,n]
FAI (%)	1.8 ± 0.5	2.2 ± 0.4	7.2 ± 1.8[b,n]
IHA	403.4 ± 6.2	435.8 ± 3.1	4479.0 ± 4.4[b,n]

a= Non-PCOS vs NA-PCOS, $p < 0.001$; b= Non-PCOS vs HA-PCOS, $p < 0.001$; c= NA-PCOS vs HA-PCOS, $p = 0.010$; d= NA-PCOS vs HA-PCOS, $p = 0.003$; e= NA-PCOS vs HA-PCOS, $p = 0.009$; f= NA-PCOS vs HA-PCOS, $p = 0.004$; G= Non-PCOS vs NA-PCOS, $p = 0.001$; h = NA-PCOS vs HA-PCOS, $p = 0.024$; i= Non-PCOS vs NA-PCOS, $p = 0.017$; j= Non-PCOS vs NA-PCOS, $p = 0.031$; k= Non-PCOS vs NA-PCOS, $p = 0.023$; l= Non-PCOS vs NA-PCOS, $p = 0.002$; m= NA-PCOS vs NA-PCOS, $p = 0.022$; n= NA-PCOS vs HA-PCOS, $p < 0.001$.

Abbreviations: BW, body weight; BMI, body mass index; DBP, diastolic blood pressure; SBP, systolic blood pressure; WC, waist circumference; WHR, waist-hip ratio; LBM, lean body mass; FM, fat mass; WTI, waist circumference-triglyceride index; CI, conicity index; BSI, body shape index; VAI; visceral adiposity index; LAP, lipid accumulation product; Go, fasting glucose; G120, glucose; Io, fasting insulin; HOMA-IR, homeostatic assessment model of insulin resistance; HOMA%B, homeostatic model assessment of β-cell function; C-pep, C-peptide; eAG, average glucose; TC, total cholesterol; HDL-C, high-density lipoprotein cholesterol; LDL-C, low-density lipoprotein cholesterol; VLDL-C, very-low-density lipoprotein cholesterol; TG, triglyceride; T, testosterone; FT, free thyroxin; DHEA, dehydroepiandrosterone; DHEAS, sulfate of dehydroepiandrosterone; A4, androstenedione; SHBG= sex hormone binding-globulin; FAI, free androgen index; IHA, index of hyperandrogenism.

 © 2022. Federação Brasileira de Ginecologia e Obstetrícia. All rights reserved.
